# Lipoxin A_4_ Preconditioning and Postconditioning Protect Myocardial Ischemia/Reperfusion Injury in Rats

**DOI:** 10.1155/2013/231351

**Published:** 2013-07-17

**Authors:** Qifeng Zhao, Lan Shao, Xingti Hu, Guowei Wu, Jie Du, Jie Xia, Huixian Qiu

**Affiliations:** ^1^Department of Cardiovascular and Thoracic Surgery, The 2nd Affiliated Hospital & Yuying Children's Hospital of Wenzhou Medical University, 109 College Western Road, Wenzhou 325027, China; ^2^Department of Cardiovascular Medicine, The 2nd Affiliated Hospital & Yuying Children's Hospital of Wenzhou Medical University, 109 College Western Road, Wenzhou 325027, China

## Abstract

This study aims to investigate the pre- and postconditioning effects of lipoxin A_4_ (LXA_4_) on myocardial damage caused by ischemia/reperfusion (I/R) injury. Seventy-two rats were divided into 6 groups: sham groups (C_1_ and C_2_), I/R groups (I/R_1_ and I/R_2_), and I/R plus LXA_4_ preconditioning and postconditioning groups (LX_1_ and LX_2_). The serum levels of IL-1**β**, IL-6, IL-8, IL-10, TNF-**α**, and cardiac troponin I (cTnI) were measured. The content and the activity of Na^+^-K^+^-ATPase as well as the superoxide dismutase (SOD), and malondialdehyde (MDA) levels were determined. Along with the examination of myocardium ultrastructure and ventricular arrhythmia scores (VAS), connexin 43 (Cx43) expression were also detected. Lower levels of IL-1**β**, IL-6, IL-8, TNF-**α**, cTnI, MDA content, and VAS and higher levels of IL-10, SOD activity, Na^+^-K^+^-ATPase content and activity, and Cx43 expression appeared in LX groups than I/R groups. Besides, H&E staining, TEM examination as well as analysis of gene, and protein confirmed that LXA_4_ preconditioning was more effective than postconditioning in preventing arrhythmogenesis via the upregulation of Cx43. That is, LXA_4_ postconditioning had better protective effect on Na^+^-K^+^-ATPase and myocardial ultrastructure.

## 1. Introduction

Myocardial ischemia/reperfusion (I/R) injury, a general health problem, is due to blood restoration after a critical period of coronary artery obstruction, which is associated with clinical interventions such as thrombolysis, angioplasty, and coronary bypass surgery. This reperfusion injury involves the activation of an inflammatory cascade and is manifested as functional impairment, arrhythmia, and accelerated progression of cell death in certain critically injured myocytes. The major mediators of reperfusion injury are oxygen radicals, calcium loading, and neutrophils [1–3]. Currently, great progress has been made in the basic research about the mechanism of myocardial I/R, but symptomatic treatment still remains as the primary therapeutic plan. With the development of molecular biology, pharmacy, and other related disciplines, it will be an important task for scientists in the future to find a safer, more effective therapeutic method.

Lipoxins (LXs) are the most recent addition to the family of bioactive products generated from arachidonic acid [4]. These molecules have both proinflammatory and anti-inflammatory actions [5] and inhibit neutrophil/granulocyte infiltration and reactive oxygen species (ROS) production [6, 7]. Lipoxins are short-lived endogenously produced nonclassic eicosanoids, whose appearance in inflammation signals the resolution of inflammation [8, 9]. Recent studies showed that LX could protect several organs such as heart, brain, lung, kidney, and stomach from I/R injury [6, 10–13]. However, there has been little research about their effect on myocardial systemic inflammation, ultrastructure changes, and arrhythmia caused by I/R injury.

Therefore, we aimed to investigate the effects of LXA_4_ preconditioning and postconditioning on myocardial I/R injury in rats. In particular, we studied the effects of LXA_4_ on oxidative stress, inflammatory reaction, Na^+^-K^+^-ATPase, and connexin 43 (Cx43) alternations caused by I/R injury.

## 2. Materials and Methods

### 2.1. Rat Model of Myocardial Ischemia/Reperfusion Injury

All animal procedures were approved by Wenzhou Medical College Animal Care and Use Committee, which is certified by the Chinese Association of Accreditation of Laboratory Animal Care (SYXK, Zhejiang 2010-0150). Sprague-Dawley (SD) male rats (eight weeks old, 200 to 250 g) were fed a standard diet and maintained in controlled environment at 25 ± 1°C under a 12 h light-dark cycle. Briefly, rats were anesthetized by an intraperitoneal injection of 10% chloral hydrate (300 mg·kg^−1^ body weight) and placed in a supine position. Blood was collected by femoral venipuncture. Next, the animals were intubated for artificial ventilation with 100% oxygen using a small animal breathing machine (tidal volume 5 mL, frequency 70 per min) and monitored by ECG. Thoracotomy was performed between the sternum and left costa, and then the pericardium was gently opened. Myocardial ischemia was induced by ligating the left anterior descending coronary artery (LAD) using a 3–0 silk suture with a section of PE tubing placed over the LAD and 1 mm from the tip of the normally positioned left atrium, and the coronary artery was occluded by pulling on the suture tightly 10 min later. After 30 min of myocardial ischemia, reperfusion started by releasing the ligature and removing the tube for 120 min. The chest wall was closed, the animal extubated, and body temperature maintained using a 37°C warm plate.

The indications of successful LAD occlusion included elevated ST segment (0.1 mv) or sharp rise of T wave, wider and higher QRS wave on ECG, and visual cyanosis of myocardial discoloration. The indications of successful reperfusion were ST segment depression (≥1/2) of the ECG, gradual falling of T wave, and QRS wave, and myocardial color turning to pink [14–16].

### 2.2. Animal Treatment and Grouping

LXA_4_ (C_20_H_32_O_5_, see Supplementary Figure 1 in the Supplementary Material available online at http://dx.doi.org/10.1155/2013/231351) was purchased from Cayman Chemical Company, Ann Arbor, USA (cat. number 90415). Seventy-two SD rats were used in the study and were randomly divided into 6 groups as follows: (1) applying LXA_4_ before I/R group 1 (LX_1_): femoral vein injection of LXA_4_ (100 *μ*g/kg, dissolved in normal saline 2 mL/kg) before the I/R procedure; (2) applying LXA_4_ after I/R group 2 (LX_2_): after 30 min of myocardial ischemia, reperfusion lasted for 30 min before LXA_4_ (100 *μ*g/kg, dissolved in normal saline 2 mL/kg) was injected through femoral vein. After that, reperfusion was resumed for an additional 90 min; (3) ischemia/reperfusion group 1 (I/R_1_): femoral vein injection of normal saline (2 mL/kg) before I/R; (4) ischemia/reperfusion group 2 (I/R_2_): after 30 min of myocardial ischemia, reperfusion lasted for 30 min, and then normal saline (2 mL/kg) was injected through femoral vein. After that, reperfusion was resumed for an additional 90 min; (5) sham group 1 (C_1_): rats underwent a similar operation without myocardial I/R, and other treatment was the same as I/R_1_ group; (6) sham group 2 (C_2_ group): rats underwent a similar operation without myocardial I/R, and other treatment was the same as I/R_2_ group.

### 2.3. Blood Collection and Tissue Harvest

Blood samples were collected in each group immediately before thoracotomy and after anaesthetization (T_1_) or after experiments (T_2_). In groups C_1_ and C_2_, T_2_ was obtained after 150 min of placing surgical suture under LAD. For all other groups, T_2_ blood samples were obtained after 120 min of reperfusion. The heart was removed after obtaining blood samples (T_2_), and a portion of myocardial tissue was fixed in 4% formalin. Pathological examination of paraffin-embedded sections was performed. A separate portion of myocardial tissue was frozen in liquid nitrogen and was kept in the freezer at −70°C.

### 2.4. Assessment of Arrhythmias

ECG was recorded throughout the experiment and at a fast speed throughout ischemia and reperfusion. Ventricular arrhythmias during ligation of LAD to reperfusion (T_3_) were determined in accordance with the guidelines of the Lambeth Convention [17]. Ventricular tachycardia (VT) was defined as a run of four or more consecutive ventricular premature beats. Ventricular fibrillation (VF) was defined as a ventricular rhythm without recognizable QRS complex, and the signal morphology changed from cycle to cycle. The ECG records for the incidence and total duration of VT and VF during reperfusion were analyzed. In order to evaluate the severity of arrhythmias, ventricular arrhythmia scores (VAS) were used by giving a certain grade to each rat as follows: 0 = less than 5 times VT; 1 = more than 5 times VT or other arrhythmias, no VF; 2 = 11~30 s VT or other arrhythmias, no VF; 3 = 31~90 s VT or other arrhythmias, no VF; 4 = 91~180 s VT or other arrhythmias, and/or less than 10 s reversible VF; 5 = more than 180 s VT or other arrhythmias, and/or more than 10 s reversible VF; 6 = irreversible VF [18].

### 2.5. Cytokine and Cardiac Troponin I (cTnI) Levels

For cytokine immunoassay, blood samples were collected by femoral venipuncture at set time points, before thoracotomy (T_1_) and after reperfusion (T_2_), and serum levels of IL-1*β*, IL-6, IL-8, IL-10, TNF-*α*, and cTnI were measured using a rat ELISA kit (Shanghai Boyun Biotech, China) in accordance with the manufacturer instructions. Cytokine levels were expressed as ng/L.

### 2.6. Malondialdehyde (MDA) Content Determination

MDA content was determined on frozen myocardial tissue by thiobarbituric acid assay kit (Nanjing Jiancheng Bioengineering Institute, China). The optical density at 532 nm was measured using a spectrophotometer (UV-2000, UNICO, Shanghai). 1,1,3,3-Tetramethoxypropane was used as an external standard. The level of MDA was expressed as nanomoles per milligram protein.

### 2.7. Superoxide Dismutase (SOD) Activity

Myocardial SOD activity was determined on frozen myocardial tissue using Xanthine Oxidase Assay Kits (Nanjing Jiancheng Bioengineering Institute, China) and expressed as units per milligram protein.

### 2.8. Na^+^-K^+^-ATPase Activity

The activity of Na^+^-K^+^-ATPase was assessed by measuring the release of inorganic phosphate (Pi) from ATP according to the kit protocol (Nanjing Jiancheng Bioengineering Institute, China). Briefly, frozen myocardial tissue was weighted and normal saline was added at a ratio of 1 : 9. Then, the homogenates were centrifuged to obtain the supernatant, followed by the measurement of protein concentration using Bradford method. Finally, Na^+^-K^+^-ATPase activity was determined by measuring the amount of inorganic phosphate with malachite green dye method and expressed as micromoles per milligram protein.

### 2.9. Histology and Immunohistochemistry

The myocardial tissue 2 mm inferior to the ligation of LAD was fixed in paraformaldehyde and embedded in paraffin. Heart tissues were cut into 5 *μ*m sections for histopathological examination by hematoxylin-eosin (H&E) staining and for immunohistochemical detection of Cx43. Briefly, rehydrated sections were pretreated with pepsin solution for 15 min at 37°C. The following protocols were according to the manual of the S-P kit (ZSGB-BIO, Beijing). The myocardium near LAD was incubated for 10 min with a peroxidase-blocking solution and goat serum solution for 30 min and then incubated with primary antibody (rabbit anti-rat Cx43) (Cell Signaling Technology, USA) overnight at 4°C. After three rinses with PBS, the sections were incubated with biotinylated secondary antibody (goat anti-rabbit IgG, included in the S-P Kit) for 30 min and further developed with 3,30-diaminobenzidine tetrahydrochloride (DAB).

### 2.10. Transmission Electron Microscopy (TEM)

For TEM examination, samples containing a 2 mm portion from the edge of the incision were immediately fixed in 0.1 M phosphate buffer containing 2.5% glutaraldehyde and 2% paraformaldehyde for 4 h. The samples were then fixed with 1% osmium tetroxide for 2 h, dehydrated through a graded ethanol series, and embedded in epoxy resin. Resin-embedded blocks were cut into 60~80 nm ultrathin sections with an ultramicrotome (PT-XL, RMC, USA). The ultrathin sections were placed on carbon coated nickel grids and examined with an H-7500 transmission electron microscope (H-7500, Tokyo, Japan) operating at 80 kV.

### 2.11. Real-Time Quantitative PCR (RT-qPCR)

Total RNA was extracted using TRIzol reagent (Invitrogen, USA) according to the manufacturer's instructions. RNA (1 ng) was reversed with the cDNA synthesis Kit (Invitrogen, USA). Real-time polymerase chain reaction (PCR) was performed using the SYBR green system (Bio-Rad, USA). Primer sequences were as shown in Supplementary Table 1. The relative quantification of a target gene was normalized to GAPDH and calculated using the absolute quantification standard curve method. Melting curve profiles were produced at the end of each PC to confirm the specificity amplifications. Each sample was analyzed in triplicate.

### 2.12. Western Blotting

Equal amounts of protein (50 *μ*g) were subjected to SDS-PAGE. Proteins were then transferred to polyvinylidene fluoride (PVDF) membrane. Membrane was blocked with 5% nonfat dry milk in Tris-buffered saline, 0.1% Tween 20 (Sigma, USA), and immunoblotting was performed using rabbit anti-rat Na^+^-K^+^-ATPase (1 : 500, Cell Signaling Technology, USA) as described by the manufacturer. Anti-*β*-Actin antibody (1 : 200, Santa Cruz, USA) was used as loading control. Blots were then developed by incubation with biotinylated anti-rabbit antibody (1 : 2000; Vector Laboratories), followed by incubation with ABC reagent (GE, USA). Signal was detected using an ECL luminescence kit (GE, USA) and X-ray film.

### 2.13. Statistical Analysis

Data were expressed as mean ± standard deviation. Statistical analysis was performed by one-way ANOVA to compare more than two groups or with paired *t*-test to compare two groups. All statistical analyses were performed using SPSS version 17.0 (SPSS Inc., Chicago, IL, USA). The significance level was set at *P* < 0.05.

## 3. Results

### 3.1. The Effects of LXA_4_ Preconditioning and Postconditioning on I/R-Induced Arrhythmias

The effects of different treatments on I/R-induced arrhythmias are shown in Table 1. In C_1_ and C_2_ groups, neither VT nor VF was observed and only few ventricular premature beats appeared during the whole procedure. In contrast, almost all rats in I/R_1_ and I/R_2_ groups experienced obvious ST segment elevation, occurring VT, and high frequency of VF. Furthermore, the frequency of arrhythmias was significantly reduced in LX_1_ and LX_2_ groups with the exception of 2 rats showing VF in LX_2_ group. Most of the arrhythmias observed occurred in the ischemia (30 min) and in the first 30 min of the reperfusion periods.

In addition, the durations of VT and VAS were markedly higher in I/R_1_ and LX_1_ compared with C_1_ group, and the same was seen for I/R_2_ and LX_2_ groups when compared with C_2_ group, (*P* < 0.05 in both cases). Finally, VAS and the VT durations of LX_1_ and LX_2_ groups were significantly (*P* < 0.05) lower than those in I/R_1_ and I/R_2_ groups. Interestingly, these parameters in LX_1_ group were significantly lower than those in LX_2_ group (*P* < 0.05), indicating that LXA_4_ preconditioning was more effective in preventing I/R-induced arrhythmias than LXA_4_ postconditioning.

### 3.2. The Effect of LXA_4_ Preconditioning and Postconditioning on Serum Levels of IL-1*β*, IL-6, IL-8, IL-10, TNF-*α*, and cTnI

**Figure 1:**
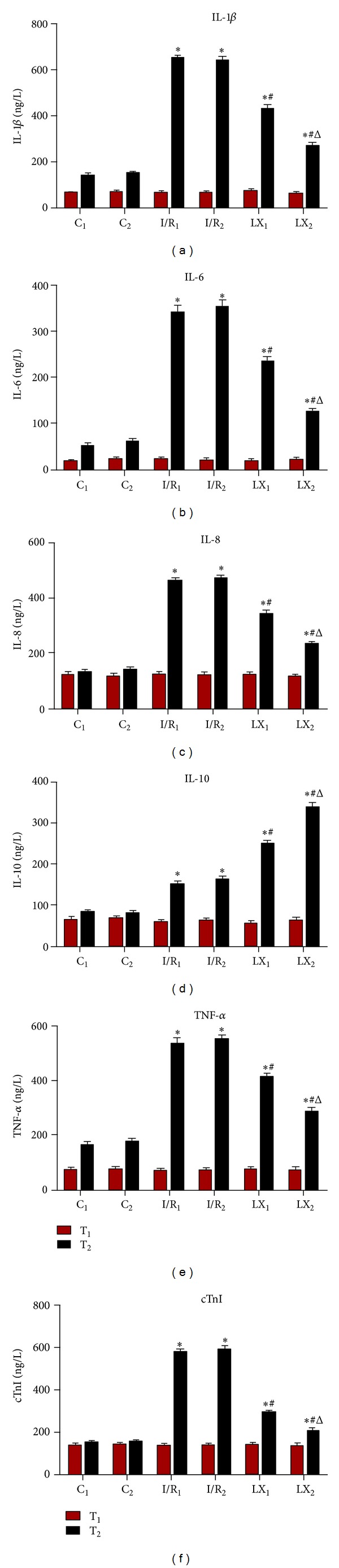
Comparison of concentrations of serum IL-1*β*, IL-6, IL-8, IL-10; TNF-*α*;, and cTnI at T_1_ and T_2_ among all groups (*n* = 12 for each group). At time point T_1_, blood was collected immediately before thoracotomy. At time point T_2_, blood was collected right after the I/R procedure was over. (a) IL-1*β*; (b) IL-6; (c) IL-8; (d) IL-10; (e) TNF-*α*; (f) cTnI. *P < 0.05 for comparisons of I/R_1_ and LX_1_ groups with C_1_ group, *P* < 0.05 for comparisons of I/R_2_ and LX_2_ with C_2_ group; ^#^ *P* < 0.05 for comparisons of LX_1_ group with I/R_1_ group, *P* < 0.05 for comparisons of LX_2_ group with I/R_2_ group; ^Δ^ *P* < 0.05 for comparisons of LX_2_ group with LX_1_ group.

Serum levels of IL-1*β*, IL-6, IL-8, IL-10, TNF-*α*, and cTnI were determined using ELISA kits. We found that there was no statistical difference at T_1_ among all the groups. In contrast, cytokines levels at T_2_ in I/R_1_ and LX_1_ groups were significantly (*P* < 0.05) elevated compared to C_1_ controls, and similar results were seen for I/R_2_ and LX_2_ groups compared with C_2_ controls. In the meantime, reduced levels of proinflammatory cytokines such as IL-1*β*, IL-6, IL-8, and TNF-*α* and elevated level of the anti-inflammatory cytokine IL-10 were observed in LX_1_ and LX_2_ groups compared with I/R_1_ and I/R_2_ groups, respectively. These findings indicate that both LXA_4_ preconditioning and postconditioning could induce the expressions of anti-inflammatory cytokines and inhibit the induction of proinflammatory cytokines. Moreover, these changes were more significant in LX_2_ compared with LX_1_ group (*P* < 0.05), further confirming that LXA_4_ postconditioning was more effective in preventing I/R injury than LXA_4_ preconditioning (Figure 1).

### 3.3. The Effect of LXA_4_ Preconditioning and Postconditioning on MDA Levels and SOD and Na^+^-K^+^-ATPase Activities

**Figure 2:**
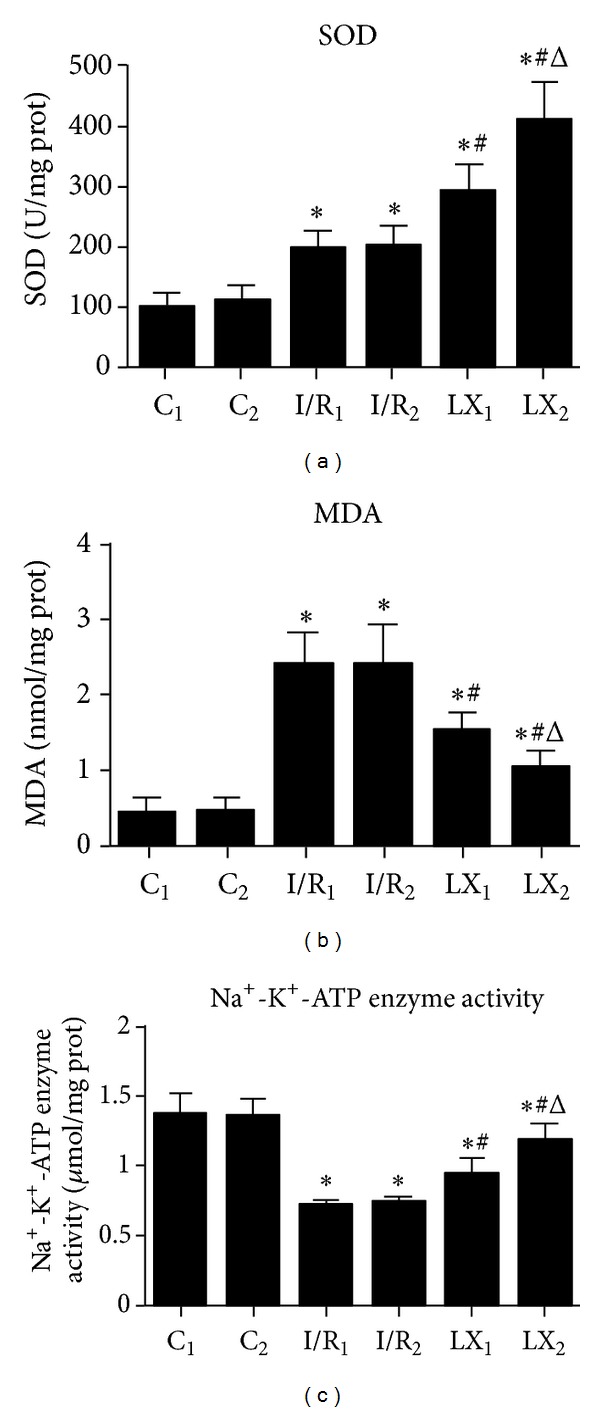
Comparison of cardiac MDA content and SOD and Na^+^-K^+^-ATPase activities at T_2_ among all groups (*n* = 12 for each group). At time point T_2_, myocardial tissue was collected right after the I/R procedure was over and kept frozen in liquid nitrogen. MDA content and SOD and Na^+^-K^+^-ATPase activities were measured as described in the section of Materials and Methods. (a) SOD activity; (b) MDA content; (c) Na^+^-K^+^-ATPase activity. *P < 0.05 for comparisons of I/R_1_ and LX_1_ groups with C_1_ group, *P* < 0.05 for comparisons of I/R_2_ and LX_2_ with C_2_ group; ^#^ *P* < 0.05 for comparisons of LX_1_ group with I/R_1_ group, *P* < 0.05 for comparisons of LX_2_ group with I/R_2_ group; ^Δ^ *P* < 0.05 for comparisons of LX_2_ group with LX_1_ group.

The production of MDA, SOD, and Na^+^-K^+^-ATPase activities in myocardial tissues in response to I/R injury were investigated. Compared with C_1_ group, MDA production and SOD activity were increased and Na^+^-K^+^-ATPase activity was reduced in I/R_1_ and LX_1_ groups, and the same results were obtained in I/R_2_ and LX_2_ groups compared to C_2_ group (*P* < 0.05). Furthermore, decreased MDA production and increased activities of SOD and Na^+^-K^+^-ATPase in LX_1_ and LX_2_ groups were observed compared with I/R_1_ and I/R_2_ groups, respectively. Similar results were observed when LX_2_ group was compared with LX_1_ group, indicating that LXA_4_ postconditioning was more protective against I/R injury than LXA_4_ preconditioning (Figure 2).

### 3.4. The Effect of LXA_4_ Preconditioning and Postconditioning on the Ultrastructural Changes of the Myocardium

**Figure 3:**
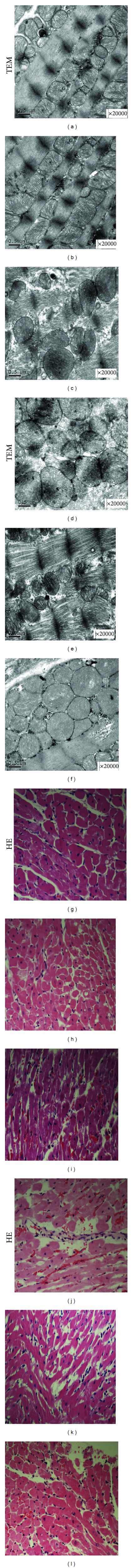
Transmission electron microscopy ((a)–(f)) and histological study ((g)–(l), He ×400) of cardiac tissues from rats of different groups at T_2_ time point. (a) Group C_1_ and (b) group C_2_: regular myocardial myofilament arrangement, clear intercalated disk structure, normal mitochondrial morphology, and structure with endoplasmic reticulum embedded in myofilament matrix; (c) group I/R_1_ and (d) group I/R_2_: disordered arrangement of myofilament, a large number of myofilament fractured, intercalated disk structure unclear, and abnormal mitochondrial morphology (a high degree of swelling, membrane lysis, fuzzy ridge structure, formation of vacuoles, and endoplasmic reticulum vacuolization); (e) group LX_1_ and (f) group LX_2_: myofilament arrangement is a little sparse, the intercalated disk structure is slightly fuzzy, and slightly swollen mitochondria; (g) group C_1_ and (h) group C_2_: normal size cardiomyocyte, no hemorrhage, or neutrophil granulocyte infiltration; (i) group I/R_1_ and (j) group I/R_2_: Cardiomyocyte degeneration, hemorrhage, edema, and significant interstitial neutrophil granulocyte infiltration; (k) group LX_1_ and (l) group LX_2_: no significant cardiomyocyte degeneration, no obvious bleeding, slight edema between the myocardial fibers, and mild neutrophil granulocyte infiltration.

TEM images of ultrathin sections of myocardial tissue are shown in Figure 3. Cardiomyocytes were clearly seen in a well-arranged myofilament and intercalated disc manner in sham groups (C_1_ and C_2_), as well as abundant normal mitochondria with no swelling, normal matrix density, and intact cristae. The round endoplasmic reticulum can also be seen between the myofilaments and the cytoplasm (Figures 3(a) and 3(b)). However, in I/R_1_ and I/R_2_ groups, myocardial I/R produced remarkable ultrastructural damages associated with irregularities and edematous separation of myofilaments, hypercontraction, and shortening of sarcomeres. Large area of cytoplasmic vacuolization and mitochondrial swelling were evident with decreased matrix density and distortion of cristae (Figures 3(c) and 3(d)). Treatment with LXA_4_ showed clear protection with relatively parallel arrangement of myofilaments and normal sarcomeres. Mitochondria were normal with mild swelling and normal matrix density but slightly damaged cristae. However, mild cytoplasmic rarefaction with mild edema could still be seen (Figures 3(e) and 3(f)).

### 3.5. The Effect of LXA_4_ Preconditioning and Postconditioning on Pathological Changes

To further assess the effect of LXA_4_ on I/R injury, we analyzed the pathological changes it induced (Figure 3). In the sham groups (C_1_ and C_2_), myocardial fibers were normal with clear striations. No epimorphosis, necrosis, or neutrophil infiltration was observed (Figures 3(g) and 3(h)). In contrast, nontreated I/R groups (I/R_1_ and I/R_2_) showed local swelling, myocardial necrosis, disorganized myocardial fibers, and ruptured cells with large number of inflammatory cells in the cytoplasm (Figures 3(i) and 3(j)). In the LXA_4_-treated groups (LX_1_ and LX_2_), myocardial cells with normal structure and shape can be seen, though the myocardial fibers were mildly swollen or partially ruptured and slight edema can be seen in the interstitial tissues with small amount of inflammatory cells (Figures 3(k) and 3(l)).

### 3.6. The Effect of LXA_4_ Preconditioning and Postconditioning on Cx43 Expression

**Figure 4:**
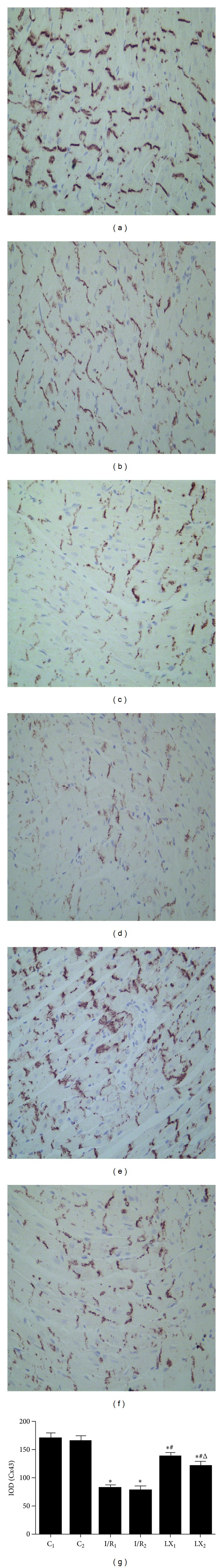
Immunohistochemical staining and IOD of Cx43 in cardiac tissues from rats of different groups at T_2_. (a) Group C_1_ and (b) group C_2_: the Cx43 positive particle intensively and regularly was distributed and was clustered in the intercalated disk; (c) group I/R_1_ and (d) group I/R_2_: sparsely distributed Cx43 positive particles; (e) group LX_1_ and (f) group LX_2_: Slightly decreased Cx43 positive particles, mostly still clustered in the intercalated disk. (g) For IOD of Cx43. *P < 0.05 for comparisons of I/R_1_ and LX_1_ groups with C_1_ group, *P* < 0.05 for comparisons of I/R_2_ and LX_2_ with C_2_ group; ^#^ *P* < 0.05 for comparisons of LX_1_ group with I/R_1_ group, *P* < 0.05 for comparisons of LX_2_ group with I/R_2_ group; ^Δ^ *P* < 0.05 for comparisons of LX_2_ group with LX_1_ group.

The gap junction protein Cx43 is the major component of gap junctions between cardiomyocytes of mammalian ventricular myocardium, including human and rodent hearts. Using an immunohistochemical approach, we wanted to know whether LXA_4_ preconditioning and postconditioning affected Cx43 expression. As shown in Figure 4, Cx43 expression, demonstrated by a pervasive brown/yellow color in cardiac muscle cells, was observed in myocardial tissue samples from all the groups. The areas of Cx43 expression in myocardial tissue samples from sham groups (C_1_ and C_2_) were larger than those detected in the I/R groups, which was also confirmed by the quantitative analysis of integral optical density (IOD) (Figure 4). Cx43 was well arranged together at both ends of the intact cardiac myocyte in I/R_1_ and I/R_2_ groups, and Cx43 staining was significantly reduced when compared with the sham groups. However, in LXA_4_-treated LX_1_ and LX_2_ groups, most Cx43 staining was located at the ends of intact cardiac myocytes (intercalate disc) and redistributed in both sides of the cell. Compared with nontreated I/R_1_ and I/R_2_ groups, the IOD value was statistically higher in LX_1_ and LX_2_ groups, respectively. Additionally, Cx43 expression of LXA_4_ preconditioning (LX_1_ group) was significantly (*P* < 0.05) higher than LXA_4_ postconditioning (LX_2_ group) (Figure 4).

### 3.7. The Effects of I/R and LXA_4_ Treatment on Na^+^-K^+^-ATPase and Cx43 at mRNA Level

**Figure 5:**
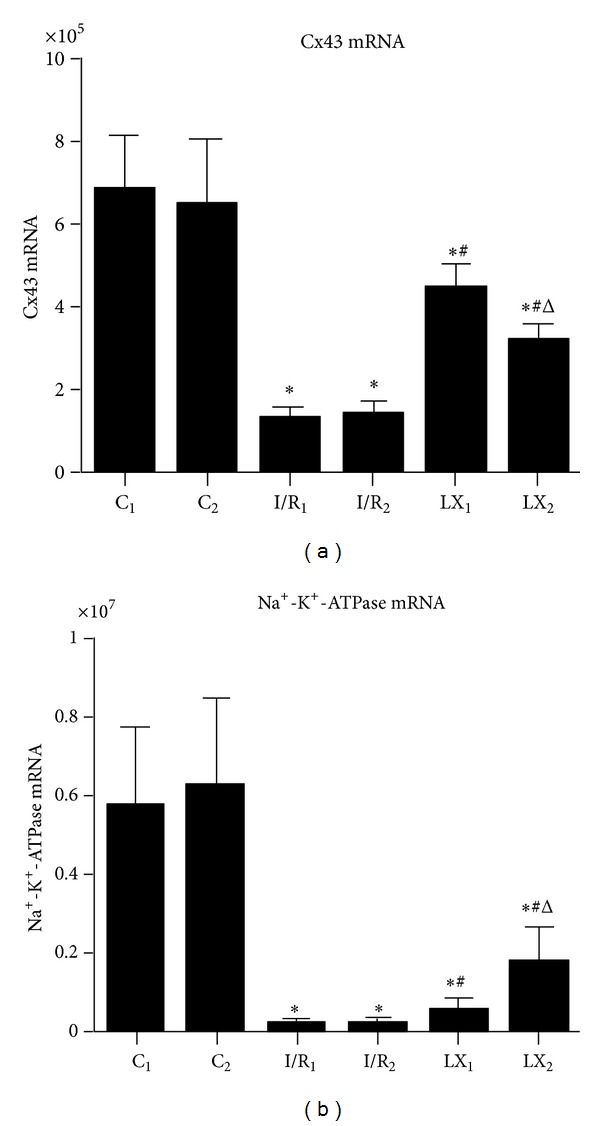
mRNA levels of Cx43 and Na^+^-K^+^-ATPase of cardiac tissues from rats of different groups at T_2_. Samples were collected right after I/R procedure. (a) Cx43 mRNA; (b) Na^+^-K^+^-ATPase mRNA. *P < 0.05 for comparisons of I/R_1_ and LX_1_ groups with C_1_ group, *P* < 0.05 for comparisons of I/R_2_ and LX_2_ with C_2_ group; ^#^ *P* < 0.05 for comparisons of LX_1_ group with I/R_1_ group, *P* < 0.05 for comparisons of LX_2_ group with I/R_2_ group; ^Δ^ *P* < 0.05 for comparisons of LX_2_ group with LX_1_ group.

The Na^+^-K^+^-ATPase and Cx43 mRNA levels were examined using real-time quantitative PCR to determine if I/R injury influences the Na^+^-K^+^-ATPase and Cx43 expressions and whether this effect is blocked by the different LXA_4_ treatment (Figure 5). Compared with the sham groups (C_1_ and C_2_), mRNA levels of Na^+^-K^+^-ATPase and Cx43 in I/R_1_, LX_1_, I/R_2_, and LX_2_ were remarkably reduced. Meanwhile, Na^+^-K^+^-ATPase and Cx43 expressions in LXA_4_-treated LX_1_ and LX_2_ groups were higher than the saline-treated I/R_1_ and I/R_2_ groups, respectively (*P* < 0.05). Furthermore, Na^+^-K^+^-ATPase mRNA level of LXA_4_ preconditioning (LX_1_) was lower than that of LXA_4_ postconditioning (LX_2_). However, Cx43 expression was higher in LX_1_ compared to LX_2_ group (*P* < 0.05).

### 3.8. Further Analysis of the Effects of LXA_4_ Treatment on Na^+^-K^+^-ATPase Isoform Expression

**Figure 6:**
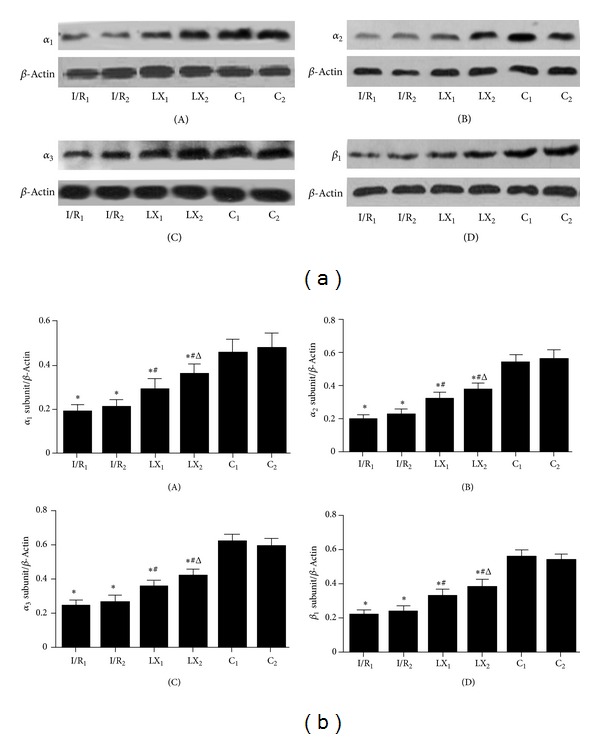
Protein levels of *α*_1_, *α*_2_, and *α*_3_ and *β*_1_ subunits of Na^+^-K^+^-ATPase of cardiac tissues from rats of different groups at T_2_. Samples were collected right after I/R procedure was over and western blot was performed as described in the section of Materials and Methods, and *β*-Actin was used as a loading control. Representative blots of three independent experiments were shown. (a) Western blotting: (A) *α*_1_ subunit of Na^+^-K^+^-ATPase; (B) *α*_2_ subunit; (C) *α*_3_ subunit; (D) *β*_1_ subunit. (b) Quantitative data of western blot: (A) *α*_1_ subunit of Na^+^-K^+^-ATPase; (B) *α*_2_ subunit; (C) *α*_3_ subunit; (D) *β*_1_ subunit. *P < 0.05 for comparisons of I/R_1_ and LX_1_ groups with C_1_ group, *P* < 0.05 for comparisons of I/R_2_ and LX_2_ with C_2_ group; ^#^ *P* < 0.05 for comparisons of LX_1_ group with I/R_1_ group, *P* < 0.05 for comparisons of LX_2_ group with I/R_2_ group; ^Δ^ *P* < 0.05 for comparisons of LX_2_ group with LX_1_ group.

The results of Na^+^-K^+^-ATPase isoforms analyzed by western blotting are shown in Figure 6. Compared to the sham C_1_ group, the expressions of Na^+^-K^+^-ATPase isoforms *α*_1_, *α*_2_, *α*_3_, and *β*_1_ were markedly reduced in I/R_1_ and LX_1_ groups and similar results were obtained in I/R_2_ and LX_2_ groups, compared to C_2_ group (*P* < 0.05). However, Na^+^-K^+^-ATPase isoform levels in LXA_4_-treated LX_1_ and LX_2_ groups were higher than the saline-treated I/R_1_ and I/R_2_ groups respectively (*P* < 0.05). Additionally, protein levels of Na^+^-K^+^-ATPase in LXA_4_ preconditioning (LX_1_) were significantly (*P* < 0.05) lower than those measured in LXA_4_ postconditioning (LX_2_).

## 4. Discussion

With the widespread use of cardiac surgery for congenital heart disease and coronary artery bypass grafting under extracorporeal circulation, thrombolytic therapy, percutaneous transluminal coronary intervention, organ transplantation, and cardiopulmonary resuscitation, it has currently become a hot topic for cardiovascular scientists to find safe and effective drugs to treat myocardial I/R injury [10]. In this study, we used rats to induce I/R injury because of the little variation in their cardiovascular system, similarity to human coronary artery territory, less myocardial collateral circulation, and the high probability of occurrence of I/R injury. Due to the smaller size of coronary artery in rats, more than 30 min of blocking time can easily lead to permanent occlusion of coronary artery [14, 19, 20]. Therefore, we chose 30 min of myocardial ischemia followed by 120 min reperfusion. LXA_4_ is usually dissolved in ethanol. However, we found that ethanol affected the health of experimental animals. Therefore, LXA_4_ was dissolved in saline as suggested by the manufacturer's manual.

Currently, the effect of I/R injury on myocardial ultrastructure is not entirely clear. El Kebir et al. [21] reported that LX can inhibit neutrophil aggregation at inflammatory sites and regulate the balance of proinflammatory/anti-inflammatory cytokines. In addition, LXA_4_ can suppress vascular-endothelial-growth-factor- (VEGF-) induced inflammatory reaction through the regulation of IL-6, TNF-*α*, IL-8, ICAM-1, and IL-10 expressions in human umbilical vein endothelial cells [22]. Souza et al. [13] found that reduced production of TNF-*α* following LXA_4_ treatment of intestinal I/R injury may be due to the increased IL-10 expression, and LXA_4_ could not prevent reperfusion injury in IL-10-deficient mice. In our study, we confirmed that I/R resulted in myocardial inflammatory injury through analyzing serum components before and after I/R. Although both proinflammatory/anti-inflammatory cytokines were upregulated, the expressions of proinflammatory cytokines were much higher than the anti-inflammatory ones. However, with LXA_4_ treatment, the expressions of proinflammatory cytokines (IL-1*β*, IL-6, IL-8, and TNF-*α*) were reduced while the anti-inflammatory cytokine IL-10 was upregulated, leading to an anti-inflammatory state (Figure 1).

The results of H&E staining confirmed that LXA_4_ could alleviate I/R injury as evidenced by reduced swelling, well-organized cardiac muscle, clear striations, and less inflammatory cell infiltration. In accordance with the pathological changes, we found that LXA_4_ protected the ultrastructures of myocardial tissue as shown by normal matrix density, relatively parallel arrangement of myofilaments, and normal sarcomere mitochondria by TEM examination (Figure 3).

There has been report showing that LXA_4_ can inhibit the generation of ROS [7]. We found that I/R groups had increased MDA levels and SOD activity, which indicated enhanced oxidative stress. After LXA_4_ treatment, SOD activity was significantly improved and MDA levels were markedly reduced (Figure 2), suggesting that the alleviated I/R injury seen after LXA_4_ treatment might be due to decreased ROS generation. Mitochondria are the main organelle maintaining the cell respiration and oxidation. Close to 90% of cellular oxygen intake is used in oxidative phosphorylation reactions in the mitochondria, but 1% to 2% of oxygen can escape to form ROS. ROS can decrease the efficiency of mitochondrial performance and cause swelling of mitochondria by attacking the mitochondrial inner membrane. Indeed, mitochondria are both a primary source and target of ROS [23]. As we can see from the TEM images (Figure 3), the swelling condition of mitochondria has been remarkably alleviated after LXA_4_ treatment, with clear cristae and well-arranged cardiac fiber ultrastructure. This agrees with the MDA levels and the SOD activity data and further confirms that reduced I/R injury may be due to the inhibition of oxygen-free radical targeting of mitochondrial membranes.

The Na^+^-K^+^-ATPase is a heterodimer that plays an important role in the maintenance of cell homeostasis by regulating membrane potential and cation transport across the sarcolemmal membrane, which is a reliable indicator of metabolic disorders and tissue injury. I/R injury could affect mitochondrial function and inhibit Na^+^-K^+^-ATPase activity, leading to intracellular calcium overload, activation of proteases, and release of oxygen-free radicals, which in turn aggravate I/R injury. Belliard et al. [24] reported that the maintenance of Na^+^-K^+^-ATPase cell surface abundance was crucial in myocyte survival after an ischemic attack treated by ouabain preconditioning, and the protection conferred by increased surface expression of Na^+^-K^+^-ATPase may be independent of ion transport. Meanwhile, Zheng et al. [25] found that the protection from cardiac injury may be determined by the Na^+^-K^+^-ATPase activity, involving ERK1/2 and PI3 K/Akt signal pathways. Furthermore, continued intermittent hypobaric hypoxia can reduce the reperfusion injury in guinea pig by increasing myocardial Na^+^-K^+^-ATPase activity [26]. The Na^+^-K^+^-ATPase consists of *α* and *β* subunits; the *α* subunit is responsible for the catalytic activity of the enzyme and includes three isoforms: *α*_1_, *α*_2_, and *α*_3_ in the rat heart. The *β* subunit is responsible for the proper localization and insertion of Na^+^-K^+^-ATPase in the sarcolemmal membrane. In this study, the expressions of *α*_1_, *α*_2_, and *α*_3_ proteins decreased after ischemia/reperfusion injury, which affected the active center and further reduced the activity of the ATPase enzyme. The expression of *β* subunit also declined after I/R injury, and this could cause damage to the cytoskeleton, lead to detachment of Na^+^-K^+^-ATPase from the cell membrane, and further reduce its activity [27]. The effect of ischemia/reperfusion on the expressions of Na^+^-K^+^-ATPase *α*_1_, *α*_2_, and *α*_3_ subunits were slightly different from earlier report [28], which may be due to differences in experimental and detection methods. The content and the activity of Na^+^-K^+^-ATPase can reflect myocardial injury to a certain extent, and I/R injury can also be alleviated by increasing its content and activity. Figtree et al. [29] suggested that cardiomyocytes could be protected potentially by reversing the oxidation inhibition of Na^+^-K^+^-ATPase. And from the our study, we know that LXA_4_ enhanced Na^+^-K^+^-ATPase subunits expressions (Figures 5 and 6) and reduced cTnI concentration, indicating that the cardioprotective effect of LXA_4_ could be due to increased Na^+^-K^+^-ATPase content and activity.

The gap junction (GJ) plays key roles not only in electrical coupling of cardiomyocytes but also in intercellular transport of biologically active substances. GJ is mainly located in the intercalated disc of the cardiac tissue [30] and participates in decision making for cell survival versus cell death in various types of cells. It has been suggested that reperfusion injury to the heart is partially mediated by gap junctions. Cx43 forms gap junctions that facilitate electrical cell-cell coupling and unopposed/nonjunction hemichannels which provide a pathway for the exchange of ions and metabolites between cytoplasm and extracellular milieu [31]. It has also been known that Cx43 participates in the process of myocardiac ischemia/reperfusion injury [32, 33]. During I/R injury, alterations in Cx43 expression, its localization and its phosphorylation status, and changes in GJ properties collectively contribute to myocardial infarction and arrhythmogenesis [34–36]. From our research, we found that Cx43 was well arranged together in both ends of intact cardiac myocytes (intercalate disc) in the sham group, while in the I/R group, Cx43 expression was reduced which was associated with damaged cellular ultrastructures (Figures 4 and 5). In addition, increased VAS, VTt, and VFt duration and reduced Cx43 mRNA and protein expressions after injury strongly indicate that I/R injury could induce arrhythmia through Cx43 pathway.

The mechanism of arrhythmogenesis caused by I/R injury has not yet been illustrated clearly. Cabo et al. found that this may be due to the structural and functional alternations of Cx43 after injury [37], and Danik reported that Cx43 knockout mice showed complicated tachyarrhythmia [38]. Recently, two lipoxins have been identified, lipoxin A_4_ (LXA_4_) and lipoxin B_4_ (LXB_4_). LXA_4_ treatment in our study showed that this molecule can alleviate Cx43 degradation and protect its distribution that limited the damage to the ultrastructure of cardiac tissue caused by I/R injury. At the same time, the mRNA transcriptional levels of Cx43 were notably increased, which was in accordance with the immunohistochemical results. This indicates that LXA_4_ plays a protective role in regulating the expressive levels of Cx43 protein and gene. In addition, reduced arrhythmia frequency and VAS were observed, indicating that the protective effect of LXA_4_ may be associated with Cx43 remodeling, an observation consistent with a previous study showing that cardiac function could be preserved through Cx43 remodeling [39].

The pharmacological preconditioning and postconditioning with LXA_4_ can both attenuate myocardial I/R injury in rats, with LXA_4_ preconditioning being more effective in preventing the arrhythmogenesis than the LXA_4_ postconditioning. An effect associated with higher Cx43 expression and lower VAS. However, LXA_4_ postconditioning had a better protective effect on Na^+^-K^+^-ATPase and the ultrastructure of the myocardium, which may be related to the physicochemical property, duration of the treatment, and the dose of LXA_4_. We also observed that most arrhythmias happened in the ischemia (30 min) and the first 30 min of reperfusion, which may be due to the activated compensatory mechanism of the injured body and increased tolerance to the drug. Cx43 is easy to be degraded when I/R injury happens [40]. Cx43 is also involved in pathological and physiological processes other than arrhythmias during I/R injury [41, 42], and it may cause uncontrolled opening of hemichannels in the plasma membrane [43] and may be deleterious to the myocardium [44]. Blocking hemichannels may confer cardioprotection by preventing ionic imbalance, excessive entry of Na^+^ and Ca^+^, ATP leakage, cell swelling, and loss of critical metabolites [40, 45, 46]. Therefore, Cx43-mediated intercellular communication of the injured tissue can be enlarged into other cells, and this may explain that LXA_4_ preconditioning induced higher Cx43 expression but caused more serious damage to myocardium. At the same time, it indicated that the up-regulation of Cx43 is helpless in the myocardial energy metabolism, systemic inflammation, and ultrastructure. The application of pharmacological preconditioning has been limited largely by the unpredictability of I/R injury, and this implicates that LXA_4_ postconditioning could have a great potential for clinical application in I/R injury in the future. 

Although there have been reports showing that LXA_4_ treatment was protective in cerebral I/R injury in rats [12] and myocardial I/R injury in mice [47] and rabbits [48], ours is the first study to report a protective effect of LXA_4_ preconditioning and postconditioning on myocardial I/R injury in rats. Further studies are needed to optimize the dose, the timing, and the route of administration of LXA_4_.

## 5. Conclusions

LXA_4_ preconditioning and postconditioning in myocardial I/R injury can attenuate the metabolic disturbance in the myocardium through upregulating Na^+^-K^+^-ATPase expression. Meanwhile, both LXA_4_ preconditioning and postconditioning protected the ultrastructure of the myocardium by inhibiting the inflammatory reaction and oxidative stress. In addition, the upregulation of Cx43 expression had a positive effect in preventing arrhythmogenesis caused by I/R injury. All the results showed that LXA_4_ has great potential for protecting the myocardium from myocardial I/R injury.

## Supplementary Material

Supplementary Figure 1: Chemical structure of LXA_4_. Lipid-derived lipoxins are produced at the site of vascular and mucosal inflammation where they down-regulate polymorphonuclear leukocyte recruitment and function. 5(S),6(R),15(R)-LipoxinA_4_ (5(S),6(R),15(R)-LXA4) is derived from the aspirin- triggered formation of 15(R)-HETE from arachidonic acid. Formula Weight: 352.5.
According to DNA sequence of various indexes, Primer 3.0 software was used to design Na^+^-K^+^-ATPase and Cx43 primer. GAPDH is control gene. Primer sequences of Na^+^-K^+^-ATPase and Cx43 were as shown in Supplementary Table 1.

## Figures and Tables

**Table 1:** The ventricular arrhythmia score (VAS), the duration time of VT (VTt) and VF (VFt) in different groups at T_3_ (*n* = 12, mean ± SD).

Group	VAS	VTt (s)	VFt (s)
C_1_	0.25 ± 0.45	0	0
I/R_1_	4.67 ± 0.49*	73.50 ± 28.40*	16.67 ± 14.62*
LX_1_	2.33 ± 0.49^∗#^	22.67 ± 7.51^∗#^	0
C_2_	0.33 ± 0.49	0	0
I/R_2_	4.83 ± 0.39*	69.67 ± 24.83*	22.00 ± 16.03*
LX_2_	3.50 ± 0.80^∗#Δ^	35.42 ± 14.92^∗#Δ^	3.58 ± 8.50^#^

Note: T_3_: during ligation of the left anterior descending coronary artery to reperfusion.

I/R_1_ and LX_1_ groups were compared with C_1_ group, and I/R_2_ and LX_2_ were compared with C_2_ group, *P < 0.05.

LX_1_ goup was compared with I/R_1 _group, and LX_2 _group was compared with I/R_2_ group, ^#^ *P* < 0.05.

LX_2_ group was compared with LX_1_ group, ^Δ^ *P* < 0.05.
