# Supplementary figures and images for: Lipoxin A4 Preconditioning and Postconditioning Protect Myocardial Ischemia/Reperfusion Injury in Rats
Source: Mediators Inflamm. 2013 Jul 17;2013:231351. doi: 10.1155/2013/231351 (PMC3730367; doi:10.1155/2013/231351)

Supplementary Figure. 1


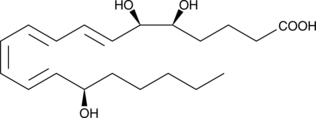


Chemical structure of LXA4

Supplement: Supplementary file 1 — Supplementary Figure 1: Chemical structure of LXA4. Lipid-derived lipoxins are produced at the site of vascular and mucosal inflammation where they down-regulate polymorphonuclear leukocyte recruitment and function. 5(S),6(R),15(R)-LipoxinA4 (5(S),6(R),15(R)-LXA4) is derived from the aspirin- triggered formation of 15(R)-HETE from arachidonic acid. Formula Weight: 352.5. According to DNA sequence of various indexes, Primer 3.0 software was used to design Na+-K+-ATPase and Cx43 primer. GAPDH is control gene. Primer sequences of Na+-K+-ATPase and Cx43 were as shown in Supplementary Table 1. [file 231351.f1.docx]
