# Supplementary material for: Lipoxin A4 Preconditioning and Postconditioning Protect Myocardial Ischemia/Reperfusion Injury in Rats
Source: Mediators Inflamm. 2013 Jul 17;2013:231351. doi: 10.1155/2013/231351 (PMC3730367; doi:10.1155/2013/231351)
Supplement: Supplementary file 2 [file 231351.f2.docx]

Supplementary Table 1 Real-time PCR primer sequences

| Gene | Forward primer | Reverse primer | Size |
| --- | --- | --- | --- |
| Na^+^-K^+^-ATPase | GCCTATCCTTAAGCGTGCAG | GCGTTTGGGTTCTTGTGAAT | 174bp |
| Cx43 | TCCTTGGTGTCTCTCGCTTT | GAGCAGCCATTGAAGTAGGC | 167bp |
| GAPDH | GAGTCAACGGATTTGGTCGT | TTGATTTTGGAGGGATCTCG | 238bp |
